# Varicose Veins: Role of Mechanotransduction of Venous Hypertension

**DOI:** 10.1155/2012/538627

**Published:** 2012-02-12

**Authors:** Hussein M. Atta

**Affiliations:** Department of Surgery, Faculty of Medicine, Minia University, Misr-Aswan Road, El-Minia 61519, Egypt

## Abstract

Varicose veins affect approximately one-third of the adult population and result in significant psychological, physical, and financial burden. Nevertheless, the molecular pathogenesis of varicose vein formation remains unidentified. Venous hypertension exerted on veins of the lower extremity is considered the principal factor in varicose vein formation. The role of mechanotransduction of the high venous pressure in the pathogenesis of varicose vein formation has not been adequately investigated despite a good progress in understanding the mechanomolecular mechanisms involved in transduction of high blood pressure in the arterial wall. Understanding the nature of the mechanical forces, the mechanosensors and mechanotransducers in the vein wall, and the downstream signaling pathways will provide new molecular targets for the prevention and treatment of varicose veins. This paper summarized the current understanding of mechano-molecular pathways involved in transduction of hemodynamic forces induced by blood pressure and tries to relate this information to setting of venous hypertension in varicose veins.

## 1. Introduction

Varicose veins are a common venous disease of the lower extremity which affects more than 30 per cent of the adult population in Western countries [1–3]. Varicose veins are elongated, dilated, and tortuous veins. Varicose veins range in severity from the undesirable appearance of telangiectasia to large tortuous varicosities with or without associated swelling, dermatitis, pigmentation, or cutaneous ulcerations [4]. There is a significant financial burden of chronic venous disease that comprises varicose veins and their complications on the health care system, with an estimated $3 billion per year being spent on the treatment of venous wounds in the United States [5]. In countries with developed health care system, the cost of treating advanced venous disease has accounted for up to 1% to 2% of the total health care budget [6, 7]. Thus varicose veins pose considerable social and economic problems.

Varicose veins are characterized by symptoms or signs produced by venous hypertension as a result of structural or functional abnormalities of veins. Symptoms may include aching, heaviness, cramps, itching, sensations of burning, swelling, dilatation or prominence of superficial veins, and skin changes. Signs may include telangiectasia, reticular or varicose veins, edema, and skin changes such as pigmentation, lipodermatosclerosis, eczema, and ulceration. A descriptive classification, known as CEAP, was developed to standardize reporting of chronic venous disorders (Table 1). The CEAP classification was based on clinical manifestations (C), etiologic factors (E), anatomic distribution of disease (A), and underlying pathophysiologic findings (P) [8, 9].

Because the history and physical examination are not always able to determine the nature and extent of venous insufficiency, a number of diagnostic investigations have been developed [10]. Currently, duplex ultrasound is the “gold standard” for venous imaging. Proper duplex ultrasound evaluation includes assessment of both reflux and obstruction in the deep, superficial, tributary, and perforating veins with precise mapping of abnormal pathways, identification of sources of reflux, accurate documentation of all target vessels to be treated [11, 12]. In normal veins, duplex examination reveals a cephalad flow phasic with respiration enhanced with distal thigh or calf compression. Valve reflux is determined by the valve closure time, the time taken for the valve to close after compression is released. Duplex scanning shows that normal valvular closure time in the standing position is less than 500 ms in the superficial veins, deep femoral vein, and perforator veins while it is less than 1000 ms in the common femoral, femoral, and popliteal veins. Reflux is considered to be present if the duration is longer [13, 14].

Treatment of superficial venous incompetence can be accomplished by different techniques including gradient elastic compression stockings, ligation and stripping, sclerotherapy, ultrasound-guided sclerotherapy, endovenous laser or radiofrequency thermal ablation, cutaneous lasers, and intense pulse light devices [15, 16].

 Varicose veins have a recurrence rate of 26% to 60% following surgery [17–19], making it important to understand the pathophysiological mechanisms involved in the development of varicose veins. Although varicose veins are relatively common, the etiology and pathogenesis of primary varicose veins remain unclear. Several proposals have been made to explain the pathogenic mechanisms involved in this disease, most notably are valvular incompetence and primary vein wall changes [20, 21]. Several risk factors have been identified to contribute to the formation of varicose veins including family history, age, sex, pregnancy, and prolonged-standing occupations (orthostatism). Perturbations in hemodynamic forces in the vein wall influence cytoskeletal organization, gene expression, proliferation, and survival and may induce inflammation and the subsequent remodeling of the wall and venous valves. All these are fundamental mechanisms that underlie various pathologies implicated in varicose veins formation.

 Because of the location of varicose veins in the lower extremity, and the increase in lower extremity venous pressure in the standing position, a relationship between the lower extremity high venous pressure and the formation of varicose veins has long been proposed. Nevertheless, the molecular pathways responsible for the transduction of the high venous pressure into vein wall dilation are not clear. Specifically, how the venous pressure could be trasduced to mechanical sensors in the vein wall, which in turn affect downstream signaling pathways and eventually affect molecular targets that modulate vein function, remains unclear. The purpose of this paper is to discuss some of the reports in the Pubmed database in order to highlight the role of mechanical transduction of venous hypertension into biochemical pathways in the vein wall and their eventual effect on downstream target molecules. We will first discuss the physiological and pathological changes in lower extremity venous pressure, the mechanosensors in the vein wall, then the signaling mechanisms and vascular mediators in the endothelium, venous smooth muscle, and extracellular matrix of the venous wall. The discussion of these signaling mechanisms and molecular targets would help in the design of new molecular targets in the management and prevention of recurrent varicose veins.

## 2. Orthostatic Venous Hypertension

**Figure 1:**
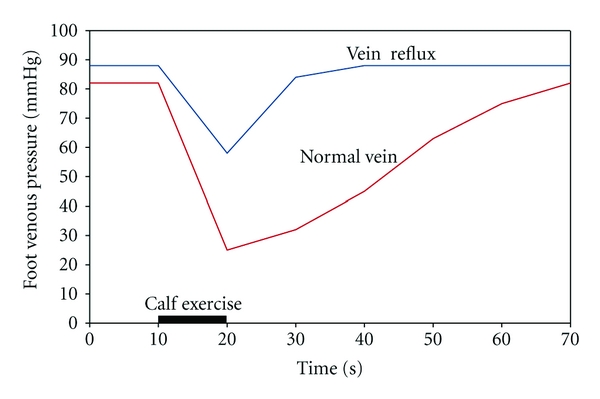
Diagram showing foot venous pressure during exercise in the standing position. Lower curve illustrates normal venous pressure while upper curve shows venous pressure in patients with venous reflux.

In the upright position and in the absence of muscle contraction, venous hydrostatic pressure measured on the dorsum of the foot ranges from 90 to 120 mmHg, depending on the individual's height [6]. Return of blood from the dependent lower extremity to the heart requires overcoming the effects of hydrostatic pressure (the weight of the column of blood below the right atrium), which is accomplished by muscle pumps working in concert with venous valves. When a normal subject exercises, the venous pressure remains at a low and steady level throughout the period of exercise [22]. However, the venous pressure measured in insufficient superficial veins during walking fell to low levels but still higher than those observed in normal veins and returned to the preexisting levels much faster when walking is stopped (Figure 1). These changes are attributed to the reflux of blood distally during muscle relaxation with the rapid establishment of the hydrostatic column and venous hypertension. The observed pressure changes at the level of the foot in normal veins are entirely dependent on intact and functioning venous valves in the distal limb. 

## 3. Mechanotransduction of Venous Hypertension

Blood vessels are constantly subjected to various types of hemodynamic forces including shear stress and circumferential stretch induced by the blood flow and pressure, respectively. Shear stress is the frictional force of the blood on the endothelial layer while the circumferential stretch is the circumferential distension of blood pressure [23]. These forces, shear stress on endothelial cells (ECs) and circumferential stretch on ECs, medial smooth muscle cells (SMCs), adventitial fibroblasts (FBs), and extracellular matrix (ECM), respectively, trigger and release biochemical mediators that maintain physiological function of blood vessels [23]. Multiple mechanosensors are involved in vascular mechanotransduction [24]. While a plethora of knowledge was gained from studies investigating mechanotransduction in arteries, little information is available regarding mechanotransduction in veins. Because venous hypertension is considered the primary underlying pathogenic mechanism in varicose vein formation and because blood pressure is translated into circumferential stretch on the vein wall, this paper will discuss how circumferential stretch is sensed and transduced in the vein wall. 

## 4. Mechanotransduction of Circumferential Stretch

**Figure 2:**
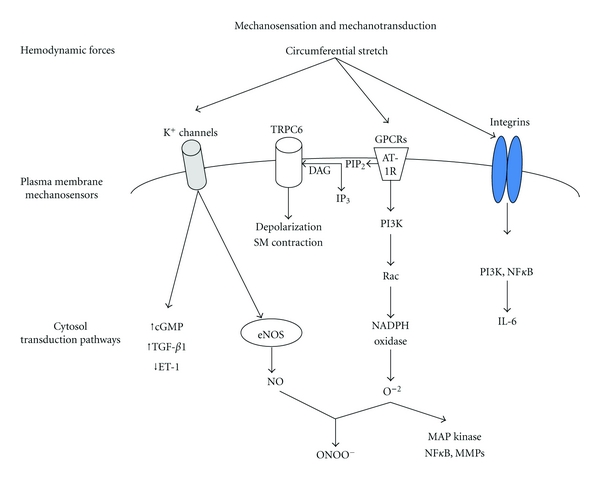
Mechanosensors of circumferential stretch in ECs. Information is compiled for both arterial and venous ECs due to scarcity of studies on venous ECs. Not all ion channels are included. This illustration is not drawn to scale.

Elevated venous pressure in the lower extremity veins during prolonged standing generates circumferential stretch of the vein wall and imposes mechanical stimulation on both ECs and SMCs. Among the most studied mechanosensing systems in response to circumferential stretch are the integrins, the flow-sensitive ion channels, and G protein coupled receptors (GPCRs) (Figure 2) [25–28]. Other candidate flow sensors that have been proposed include the glycocalyx and the platelet endothelial cell adhesion molecule-1/cadherin/vascular endothelial growth factor receptor-2 complex [23, 29–34].

## 5. Integrins

Integrins are a large family of cell surface receptors that provide adhesion of cells to both the extracellular matrix (ECM) and neighboring cells. The ability of integrins to act as a bridge between the extracellular environment and cytoskeleton/signal protein kinases enables them to transmit inside-out and outside-in signals and to mediate both bidirectional force transmission and signal transduction via an allosteric mechanism by switching between active and inactive conformations [35, 36]. Each integrin receptor is a noncovalently linked heterodimer composed of an *α* subunit and a *β* subunit. There are at least 18 *α* subunits and 8 *β* subunits, and these subunits associate to generate at least 24 different integrins [37, 38]. Of the 24 known integrins, 16 have been reported to have involvement in some aspect of vascular biology. Integrins reported to be expressed in ECs and VSMCs are depicted in Table 2 [39–42].

Several studies have demonstrated the role of integrin as mechanotransducers of circumferential stretch in ECs and SMCs. Meng et al. studied the effect of a single longitudinal stretch on human saphenous vein and examined the expression of integrin *α*_*v*_, matrix metalloproteinase (MMP)-2 and pro-MMP-9, key molecules in vascular remodeling. They found that, at peak expression on day 3, stretched vein contained 206 ± 18%  *α*_*v*_ compared to nonstretched veins. Stretched veins also secreted 177 ± 16% active MMP-2 and 161 ± 36% pro-MMP-9 [43]. Several integrins such as *α*_2_, *α*_5_, *β*_1_, and *α*_*v*_ *β*_3_ have been identified in SMCs derived from human saphenous vein [44]. On the cellular level, Sasamoto et al. demonstrated that mechanotransduction by integrin is essential for IL-6 secretion from human umbilical vein endothelial cells (HUVECs) in response to uniaxial continuous stretch. The mechanical stretch activates integrin which in turn activates NF-*κ*B in a phosphatidylinositol 3-kinase- (PI3-kinase-) dependent manner (Figure 2) [35]. 

 The role of integrins as mechanotransducers has also been reported in studies on cardiac and arterial SMCs. In VSMCs from rat heart, cyclic strain was found to stimulate mitogenesis through integrin *α*_*v*_ *β*_3_-dependent release of platelet-derived growth factor [45]. In arterial SMCs, integrin signaling is an integral element of the response to the mechanical stress [23, 46]. Circumferential stretch induces integrin activation through interactions with the ECM [23, 47]. Downstream integrin signals to Rho kinase and focal adhesion kinase (FAK) phosphorylation pathways [23, 48]. Integrin activation is also involved in Src-family tyrosine kinase activation by the direct protein-protein interactions [49]. Activated Src kinase can further activate FAK by phosphorylation. Paxillin is the target of activated FAK for phosphorylation in addition to multiple other kinases. The phosphorylated paxillin is involved in coordinating the regulation of the cell's motile machinery and thus in cell migration and survival [50]. 

## 6. Flow-Sensitive Ion Channels 

Activation of flow-sensitive ion channels is one of the most rapid EC responses to flow [25]. Several types of ion channels in the ECs have been described including K^+^ channels, Ca^2+^-activated K^+^ channels, Cl^−^ channels, Ca^2+^ channels, and Na^+^ channels [24, 25, 51]. Flow-sensitive ion channels provide ECs with the ability not only to sense flow but also to distinguish among and respond differently to different types of flow [24]. The relevance of flow-sensitive ion channels in modulating EC susceptibility to inflammation induced by low or oscillatory shear stress, a common flow type in dilated veins with functional reflux, is evident from the following discussion. It has been shown that low and oscillatory flow are equally effective in activating flow-sensitive K^+^ channels, whereas Cl^−^ channels are activated by steady flow but not oscillatory flow [52]. In bovine aortic endothelial cells (BAECs), pharmacological blockers of flow-sensitive K^+^ channels attenuate important endothelial responses to flow including release of both cGMP [53] and NO [54], upregulation of transforming growth factor beta 1 (TGF-*β*_1_) [55], and endothelial nitric oxide synthase (eNOS) [56] mRNA levels, and downregulation of endothelin-1 protein expression (Figure 2) [25, 57]. Several studies have demonstrated the involvement of ion channels in venous dilatation and varicose vein formation. Raffetto et al. showed that MMP-2 caused relaxation of rat inferior vena cava (IVC) circular segment by activation of two types of K^+^ channels, the ATP-sensitive K^+^ channel (K_ATP_) and large conductance Ca^2+^-activated K^+^ channel (BK_Ca_) [58]. Recently, Xia et al. showed in rat IVC a reduced Ca^2+^ entry via voltage-gated Ca^2+^ channels in female compared with male. In this study the authors found that the reduced contraction, Ca^2+^, and Ca^2+^ sensitivity in female veins render them more prone to dilation. These sex-specific reductions in venous function, if they also proved to occur in human veins, may explain the greater incidence of varicose veins in females [59]. Although these studies demonstrate that ion channels could be involved in varicose vein formation, their activation was not flow-mediated and thereby cannot be considered due to mechanotransduction of venous pressure. 

## 7. G Protein-Coupled Receptors (GPCRs) 

It has been shown that angiotensin II type 1 receptor (AT1R), a member of the GPCR family, is widely expressed in the vasculature. Although the following discussion is based on experimental studies of GPCR in arteries, the existence of myogenic response in human saphenous veins and the recent discovery of involvement of GPCR in this physiologic response (see below) would support a possible link between GPCR as a mechanotransducer and varicose vein development. This assumption needs to be proved by future studies. AT1R can be activated directly by circumferential stretch in the absence of a ligand, that is, independent of angiotensin II [60], leading to an active AT1R receptor conformation [61]. AT1R activation induces vascular constriction and upregulates NADPH oxidase activity and enhances superoxide production resulting in ECs and SMCs dysfunction. The increase in superoxide production by vascular cells caused by stretch has been implicated in activation of NF*κ*B [62], activation of MMPs [63], MAP kinase activation [64], angiogenesis [65], and altered vasomotion [66]. The downstream signals from AT1R include Rac activation via PI3-kinase activation (Figure 2) [23, 67]. 

Another mechanism by which GPCRs act as mechanosensors is in cooperation with transient receptor potential ion channels (TRPCs). Schnitzler et al. show that GPCRs, including AT1R, are essential mechanosensing components in VSMCs and function as sensors of membrane stretch leading to TRPC activation [26]. Stretch induces activation of the trimeric G proteins. The activated GPCRs lead to activation of phospholipase C (PLC), which hydrolyses the membrane phospholipids phosphatidyl inositol 4,5-bisphosphate (PIP_2_) into diacylglycerol (DAG) and inositol 1,4,5-trisphosphate (IP_3_). TRPC channels have been shown to be activated by PLC [68], possibly through direct actions of DAG [69, 70] generated through PLC activity [26]. TRPC activation results in Ca^2+^ influx and smooth muscle contraction (Figure 2) [71]. Although these pathways were reported in cerebral and renal arteries, the same or similar paradigm may be operative in veins exhibiting myogenic response. Studies have shown that saphenous vein of humans and animals exhibits an active myogenic response with vasoconstriction when subjected to increased intraluminal pressure [72, 73]. It is plausible to speculate that dysfunction of the AT1R-mediated activation of TRPC would lead to loss of the myogenic response (vein wall vasoconstriction) to the high venous pressure with subsequent vein wall dilation. 

## 8. Stretch Signaling Pathways in Varicose Veins 

**Figure 3:**
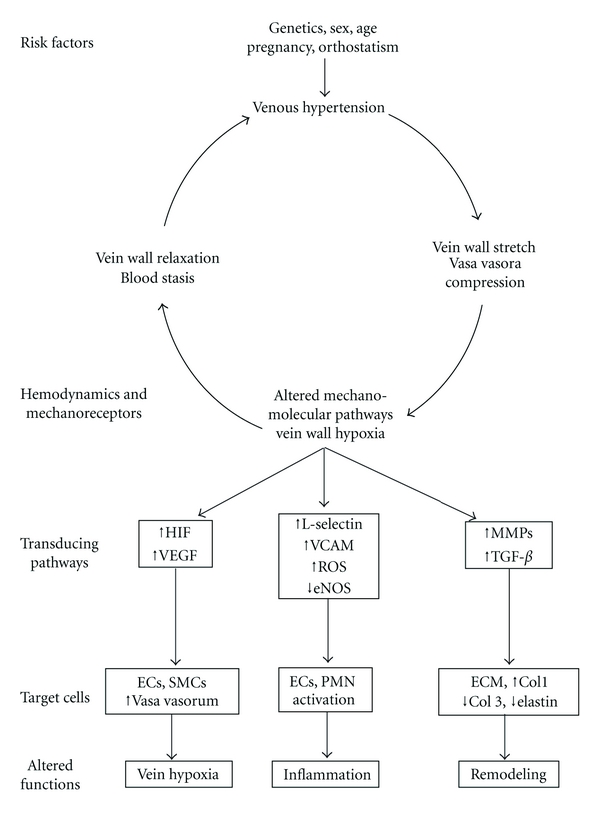
Signaling pathways of varicose vein formation.

Although the link between mechanosensors translation of circumferential stretch to biological molecules remains to be fully elucidated, considerable knowledge of downstream signaling pathways has been gained. The multitude of signaling pathways involved in varicose vein formation can, however, be grouped under three mechanisms, vein wall hypoxia, inflammation, and remodeling (Figure 3). 

### 8.1. Stretch-Induced Hypoxia 

Hypoxia of the lower extremity vein wall has been proposed as a causative factor in varicose vein formation [74–76]. Oxygen and nutrients are supplied by diffusion from the vein lumen to the endothelial and inner third of the media and from vasa vasorum to the outer two-thirds of the media [74, 77]. Venous hypertension and blood stasis both contribute to hypoxia of the varicose vein wall. Endoluminal hypoxia due to stagnation of venous blood flow results in reduced oxygen replenishment in comparison to normal venous flow. Endothelial and inner layers of the vein wall are mainly affected by endoluminal hypoxia [78]. Distension of the vein by hydrostatic pressure secondary to blood stasis causes compression of vasa vasorum leading to hypoxia of the media and outer layers of the vein wall [79]. 

 Several studies have measured the oxygen content of venous blood carried in varicose and nonvaricose veins. Unfortunately, the findings from these studies have been inconsistent and inconclusive due to variation of sampling (site, posture, type of control vein) and method of oxygen measurement [74]. On the other hand, only one study measured and compared vein wall oxygen tension between varicose and non-varicose veins in vivo. In this study, the authors reported that the average minimum oxygen tensions were significantly lower in the media of varicose compared to non-varicose veins (7.9 versus 13.4 mmHg; *P* < 0.05) [76]. Taken together, it can be concluded that several factors act synergistically to accentuate vein wall hypoxia. Venous hypertension produces stretch of the vein wall which causes increased vein wall oxygen demand and compression of vasa vasorum; both lead to vein wall hypoxia. Vein wall hypoxia in turn leads to vein relaxation [80] leading to blood stasis, which accentuates venous hypertension, and increased vein wall tension, and so on. This self-perpetuating cycle continues as long as venous hypertension is maintained [74]. 

 As hypoxia of the wall of the vein continues the number of vasa vasora should also increase in number in order to provide nutrition and oxygen to the dilated hypoxic vein wall. Histological examinations of varicose veins have consistently demonstrated an increase in the number of vasa vasorum in the wall of varicose veins particularly in the hypertrophic portions in comparison to the atrophic portions of varicose veins or to normal veins [81, 82]. It is well known that the proangiogenic cytokine, vascular endothelial growth factor (VEGF), and its upstream transcription factor hypoxia inducible factor-1 alpha (HIF-1*α*) play an important role in new vessel formation such as vasa vasora (Figure 3) [83, 84]. The role of VEGF in varicose veins was reported in several publications. It has been demonstrated that both VEGF protein and receptor and the transcription factor HIF-1*α* are elevated in the wall of varicose veins in comparison with normal veins [85, 86]. The plasma levels of VEGF have been shown to increase during the venous hypertension induced by 30 min of standing in both normal subjects and patients with varicose veins. Both supine and standing VEGF levels are higher in patients than in normal controls [87]. Moreover, plasma VEGF levels are higher in varicose vein patients with skin changes than in varicose vein patients with normal skin [88]. Finally, Howlader and Coleridge measured plasma levels VEGF using ELISA in 108 patients with varicose veins of different clinical stages and in 30 volunteers in order to determine basal VEGF levels and to explore the relationship between symptoms attributable to venous disease and VEGF. The study showed that there was a trend towards raised VEGF in all stages of varicose veins, but this only reached statistical significance in those with healed ulceration. The symptom of swelling was associated with raised VEGF levels; however, the symptoms of heaviness, cramps, and paresthesias were not associated with raised VEGF levels [89]. 

 Several animal studies supported the role of stretch-induced HIF/VEGF in the increase of vasa vasorum in the wall of varicose veins. Milkiewicz et al. found that HIF-1*α* and HIF-2*α* were elevated in stretch-induced but not shear-stress-induced angiogenesis. Rat extensor digitorum longus muscles were overloaded to induce stretch or exposed to the dilator prazosin to elevate capillary shear stress, and capillaries from these muscles were isolated by laser capture microdissection for RNA analysis. HIF-1*α* and HIF-2*α* transcript levels increased after 4 and 7 days of stretch, whereas a transient early induction of HIF-1*α* and HIF-2*α* transcripts was detected in capillaries from prazosin-treated muscles. Skeletal muscle microvascular endothelial cells exposed to 10% stretch *in vitro *showed an elevation in HIF-1*α* and HIF-2*α* mRNA, which was preceded by increases in HIF-binding activity. Conversely, HIF-1*α* and HIF-2*α* mRNAs were reduced significantly, and HIF-*α* proteins were undetectable, after 24 h exposure to elevated shear stress. Together, these results illustrate that activation of HIF-1*α* and HIF-2*α* contributes significantly to stretch- but not to shear-stress-induced angiogenesis [90]. Recently, Lim et al. reported that HIF-1*α* and HIF-2*α* mRNA were overexpressed in segments of rat IVC exposed to prolonged 2-g tension and that the overexpression was reversed by the HIF inhibitor U0126 [91]. Therefore, from the above clinical and experimental studies one can speculate that distention of the vein by the elevated hydrostatic pressure secondary to venous stasis causes compression of vasa vasorum leading to vein wall hypoxia. Vein wall hypoxia and stretch induce upregulation of HIF-1 expression. HIF-1 causes elevation of VEGF, MMP-2 and MMP-9 expression. VEGF stimulates angiogenesis and the increase in the number of vasa vasorum. MMP-2 and MMP-9 degrade collagen 3, produce vein dilation, and activate of TGF-*β*. TGF-*β* activates genes responsible for tissue remodeling such as connective tissue growth factor (CTGF) leading to increased collagen 1 and fibronectin production. 

### 8.2. Stretch-Induced Inflammation

Evidence has accumulated to support the role of venous hypertension and shear stress in the development of inflammation both of the wall of the veins and in the surrounding dermal and subdermal tissues [92]. It is generally agreed that laminar shear stress promotes the release of factors that suppress inflammation and reactive free radicals production, while low shear stress, turbulent flow, and stasis promote the production of inflammation and thrombosis [4]. Although it is not known what initiates the inflammatory events in the vein wall, it is generally accepted that leucocyte-endothelial interaction is the underlying mechanism. ECs are activated by hypoxia or by altered shear stress [78]. Leucocytes are also activated by altered shear stress and venous hypertension [93, 94]. Studies have shown that the plasma markers of leucocyte activation and leucocyte-endothelial interaction (L-selectin, intercellular adhesion molecule (ICAM)-1, endothelial leukocyte-adhesion molecule (ELAM)-1, vascular-cell adhesion molecule (VCAM)-1 are increased after subjecting patients with varicose veins to 30 minutes of standing to induce venous hypertension (Figure 3) [94, 95]. Also plasma from patients with varicose veins induces more oxygen free radical production and pseudopod formation in normal, quiescent leukocytes indicating their activation than does plasma from control subjects [96]. Neutrophil leucocyte activation was also demonstrated by the finding that plasma from patients with varicose veins contains higher levels of lactoferrin and neutrophil elastase than in age- and sex-matched controls. These enzymes are released from neutrophil granules and are considered markers of neutrophil activation [97]. In addition, monocytes/macrophages, lymphocytes, and mast cells have all been detected in the wall as well as valves of varicose veins [98, 99]. Moreover, it was demonstrated that activation was restricted not only to the luminal venous endothelium but also the endothelium in the vasa vasora of refluxing saphenous veins, as indicated by the upregulation of ICAM-1 [100]. A recent study has reported the expression of proinflammatory cytokines and chemokines in patients with varicose veins. This study found a marked expression of monocyte-chemoattractant protein (MCP)-1, macrophage-inflammatory protein (MIP)-1*α*, MIP-1*β*, interferon-*γ*-inducible protein (IP)-10, interleukin (IL)-8, and RANTES in varicose veins, suggesting that chemokines might play an important role in their pathophysiology by recruiting leucocytes to the vein wall and contributing to inflammation and edema. It is known that MCP-1, MIP-1*α*, MIP-1*β*, and IP-10 are chemoattractants for monocytes/macrophages, IL-8 is able to attract neutrophils, and RANTES is a chemotactic molecule for leucocytes [101]. Taken together, there is ample evidence of a role of inflammation in varicose vein formation and that inflammation is produced at least in part by venous hypertension. It remains, however, to delineate the molecular mechanism by which venous hypertension produces inflammation and whether this mechanism involves mechanotransduction of the altered shear stress and circumferential stretch operative in varicose veins. 

 Superoxide production has been shown to contribute to vein wall inflammation and dysfunction. It has been shown that sustained stretch increases O^−2^ production by both cultured ECs and SMCs [62, 102]. Stretch results in angiotensin II (Ang II) release from the ECs which acts on AT1R to enhance the activity of NADPH oxidase leading to higher superoxide production [23, 103]. Superoxide scavenges nitric oxide (NO) and therefore abolishes its vasoprotective effects [23]. Thus increased stretch and its subsequent stimulation of vascular oxidant stress may contribute to the cellular dysfunction encountered in the wall of varicose veins (Figure 3) [62]. Interestingly, Sohn et al. demonstrated that Ang II was shown to activate Ang II receptor type 2 (AT2R) which antagonizes AT1R-mediated superoxide formation in endothelial cells by a pathway involving tyrosine phosphatases [104]. Additionally, AT2R enhances eNOS phosphorylation via bradykinin receptors and elevates NO generation [105]. The relative distribution of both receptor subtypes on the same cell type, which may vary following prolonged venous hypertension, may therefore influence endothelial superoxide formation and hence NO dependent dilation.

### 8.3. Stretch-Induced Vein Wall Remodeling

#### 8.3.1. Role of Collagen and Elastin

Several studies demonstrated that there is significant hypertrophy of the media layer of the wall of varicose veins compared with normal veins [106]. Other studies showed that the medial layer consisted of increased number of collagen fibres and that elastin fibres are constantly fragmented with paralleled interruption of the internal elastic lamina. The adventitial layer showed decreased density and size of elastin fibres and increased fibre degradation [107, 108]. Several of the changes observed in connective tissue are thought to reflect variations in the levels of MMPs, the enzymes involved in regulating and maintaining the extracellular matrix that degrade extracellular matrix molecules, proteoglycans, elastin, and different types of collagens [109]. 

 Biochemical studies have also demonstrated a decrease in the elastin content of the affected vein wall [110]. Pascual et al. recently demonstrated that the reduction in elastin in the varicose condition may be related to the decreased lysyl oxidase-like 1 (LOXL1) levels observed. LOXL1 is a cross-linking enzyme responsible for elastin polymer deposition. These events could reduce spontaneous reticulation of elastin and the partial loss of tissue elasticity in this group of patients [111]. 

 Collagen dysregulation in varicose veins have also been the subject of several studies. Collagen type I mRNA expression and protein synthesis are increased in tissue media of varicose veins due to gene overexpression [112, 113]. Collagen III production in SMCs from varicose veins, however, was shown to be decreased due to extracellular degradation by a mechanism involving MMPs (Figure 3) [114]. This imbalance between the synthesis of collagen type I and collagen type III could explain the lack of elasticity of varicose veins [112]. 

 In a recent investigation that examined the link between the loss of ECM and the biophysical properties of varicose veins, Jeanneret and colleagues identified that adventitial elastin and intimal type III collagen were specifically and differentially affected. The selective loss of these two elastic structural proteins can explain two biophysical properties of the vein wall. Venous adventitial elastin has a critical role in regulating the venous diameter at rest, and, therefore, elastin loss may be a key factor in the development of varicose veins, preceding and precipitating dilatation. The concomitant reduction in intimal type III collagen is involved in abnormal distensibility [115]. 

#### 8.3.2. Role of MMPs

The relation between venous stretch and MMP expression in the wall of varicose veins has been extensively investigated. In a series of studies to clarify the relationship between MMP and vein wall dilatation, Raffetto et al. showed that MMPs exert vasoactive effect on vein wall. They demonstrated that MMP-2 (1 mg/mL) caused relaxation of phenylephrine-contracted rat IVC segments by a mechanism involving hyperpolarization and activation of K^+^ channels [58]. Another study from the same group was performed in rat IVC to test the hypothesis that prolonged increases in vein wall tension cause overexpression of MMPs and decreased contractility, which in turn promotes venous dilation. The results demonstrated that increases in magnitude and duration of wall tension were associated with reduced contraction and overexpression of MMP-2 and MMP-9. There was a direct correlation between the expression of MMP-2 and MMP-9 and decreased vein contractile function [116]. More recently, they reported the results of a study evaluating rat IVC stretch in the induction of HIF and MMPs. The main study findings were that HIF-1*α* and HIF-2*α* mRNAs were overexpressed in IVC exposed to prolonged 2-g tension, and the overexpression was reversed by the inhibitors of HIF. In addition, the overexpression of HIF-1*α* and HIF-2*α* in stretched IVC was associated with increased MMP-2 and MMP-9 mRNAs and proteins. The authors concluded that prolonged increases in vein wall tension are associated with overexpression of HIF-1*α* and HIF-2*α*, increased MMP-2 and MMP-9 expression, and reduced venous contraction in rat IVC. This study elucidated the mechanism and relation of HIFs and MMPs indicating that increased vein wall tension induces HIFs overexpression and causes an increase in MMPs expression and reduction of venous contraction, leading to progressive venous dilation which may contribute to varicose vein formation (Figure 3) [91]. 

#### 8.3.3. Role of TGF-*β*_1_

Several studies have demonstrated that mechanical stretch can activate latent TGF-*β*_1_ present in the ECM and that this process is inhibited by antagonizing integrins and independent from protease activity, thus establishing a link between the mechanical force of vein wall stretch and activation of the biological molecule, TGF-*β*_1_ [117–120]. TGF-*β*_1_ is a multifunctional cytokine that regulates diverse cellular functions such as proliferation, migration, differentiation, and extracellular matrix production. 

 Clinical investigations have demonstrated increased expression of TGF-*β*_1_ and its downstream signaling proteins in the wall of varicose veins compared to normal veins (Figure 3) [82, 121, 122]. Jacob et al. investigated the expression and correlation of TGF-*β*_1_ and loss of vascular SMCs, in a series of normal and varicose vein specimens. They found that TGF-*β*_1_ was differentially overexpressed in varicose veins with greater expression in the tortuous segments compared with the nontortuous segments of the varicose vein. Only a very small amount of expression of TGF-*β*_1_ was detected in normal vein samples. Histological examination revealed disruption of elastic lamellae and a significantly high degree of loss of vascular SMCs [81]. It has been shown that TGF-*β*_1_ acts as a bifunctional regulator of SMC proliferation, apoptosis, migration, and phenotype [81, 123]. O'Callaghan and Williams have shown that exposing human SMCs cells to 5 days of chronic cyclical mechanical strain increased fibronectin and collagen concentrations and MMP-2 activity and TGF-*β*_1_ expression when compared with cells grown in static conditions [124]. 

 These studies showed that mechanical stress is responsible for overexpression of TGF-*β*_1_ and for activation of its latent inactive form that already exist in the ECM. Moreover, it has also been demonstrated that TGF-*β*_1_ mediates tissue remodeling through regulation of collagen synthesis, MMP synthesis, and differentiation of fibroblasts into contractile myofibroblasts.

## 9. Conclusions and Perspectives 

The pathobiology of varicose vein formation is one of the fields that need increasing research attention given the significant social, medical, and economic importance. The recent characterization of several mechanosensors in the ECs and SMCs will stimulate future research of the mechanotransduction signaling pathways involved in the development of varicose veins. Possible areas of future research and intervention could be suggested based on current understanding of mechanotransduction. One such research areas would investigate stretch-induced myogenic response in human saphenous vein. Given that studies have shown that saphenous vein of humans and animals exhibits an active myogenic response and that stretch-induced AT1R and TRPC channels signaling mechanism are responsible for the myogenic response in arteries, it remains, however, to delineate the underlying signaling pathways and the identity of mechanosensors of myogenic response in human superficial veins of the lower extremities. Failure of this myogenic response may have a critical role in the development of varicose veins. A second area of research would be confirming in nonvaricose and varicose saphenous veins the previous animal studies that shows reduced contraction in female rat IVC compared with male due to reduced Ca^2+^ entry via voltage-gated Ca^2+^ channels. This would explain the greater incidence of varicose veins in females. A third topic would be investigating the relevance of flow-sensitive ion channels, such as flow-sensitive K^+^ channels, in modulating venous ECs susceptibility to inflammation induced by low or oscillatory shear stress in human varicose veins. Future studies should also confirm and expand on previous findings showing that a single longitudinal stretch of saphenous vein grafts produces significant increase in the expression and activation of *α*_*v*_ integrin, MMP-2, and MMP-9, key molecules in vascular remodeling. These and other studies would expand our understanding on how vein wall ECs, SMCs, FBs, and ECM sense and transduce stimuli of hemodynamic forces, particularly venous hypertension, into biochemical signals. This knowledge would be critical to developing new therapies for the prevention and treatment of varicose veins. 

## Figures and Tables

**Table 1:** The CEAP classification.

Clinical classification (C)	
C_0_	No visible or palpable signs of venous disease
C_1_	Telengiectases and/or reticular veins
C_2_	Varicose veins
C_3_	Edema
C_4*a*_	Pigmentation and/or eczema
C_4*b*_	Lipodermatosclerosis and/or atrophie blanche
C_5_	Healed ulcer
C_6_	Active ulcer
C_*s*_	Symptoms, including ache, pain, tightness, skin irritation, heaviness, muscle cramps
C_*A*_	Asymptomatic

Etiologic classification (E)	

E_*c*_	Congenital (Klippel-Trenaunay syndrome)
E_*p*_	Primary
E_*s*_	Secondary (post-thrombotic syndrome, trauma)
E_*n*_	No venous cause identified

Anatomic classification (A)	

A_*s*_	Superficial
A_*d*_	Deep
A_*p*_	Perforator
A_*n*_	No venous location identified

Pathophysiologic classification (P)	

P_*r*_	Reflux
P_*o*_	Obstruction, thrombosis
P_*r*,*o*_	Reflux and obstruction
P_*n*_	No venous pathophysiology identified

**Table 2:** Integrins expressed in ECs and VSMCs.

ECs [39, 40]	VSMCs [41, 42]
*α*_1_ *β*_1_	*α*_1_ *β*_1_
*α*_2_ *β*_1_	*α*_2_ *β*_1_
*α*_3_ *β*_1_	*α*_3_ *β*_1_
—	*α*_4_ *β*_1_
*α*_5_ *β*_1_	*α*_5_ *β*_1_
*α*_6_ *β*_1_	*α*_6_ *β*_1_
*α*_6_ *β*_4_	*α*_6_ *β*_4_
—	*α*_7_ *β*_1_
—	*α*_8_ *β*_1_
—	*α*_9_ *β*_1_
—	*α*_*v*_ *β*_1_
*α*_*v*_ *β*_3_	*α*_*v*_ *β*_3_
*α*_*v*_ *β*_5_	*α*_*v*_ *β*_5_
